# Excess Burden of Poverty and Hypertension, by Race and Ethnicity, on the Prevalence of Cardiovascular Disease

**DOI:** 10.5888/pcd20.230065

**Published:** 2023-11-23

**Authors:** Michael L. Sells, Ethan Blum, Geraldine S. Perry, Paul Eke, Letitia Presley-Cantrell

**Affiliations:** 1 Division for Heart Disease and Stroke Prevention, National Center for Chronic Disease Prevention and Health Promotion, Centers for Disease Control and Prevention, Atlanta, Georgia; 2 ASRT, Inc, Smyrna, Georgia; 3 Division of Population Health, National Center for Chronic Disease Prevention and Health Promotion, Centers for Disease Control and Prevention, Atlanta, Georgia

## Abstract

**Introduction:**

Cardiovascular disease (CVD) is the leading cause of death in the United States. Certain demographic characteristics are associated with disparities in CVD and its risk factors, which may interact with specific social determinants of health (SDOH). We examined the association of a single SDOH (ie, poverty level) with diagnosed CVD morbidity and the joint influence of poverty and hypertension on the prevalence of CVD morbidity among non-Hispanic Black, non-Hispanic White, and Hispanic people aged 30 years or older.

**Methods:**

We used data from the National Health and Nutrition Examination Survey collected during 1999 to 2018. We assessed the prevalence of diagnosed CVD morbidity (eg, self-reported coronary heart disease, angina, myocardial infarction, or stroke) by using a Poisson family with a log link regression model. We calculated the additive interaction of poverty level with hypertension on diagnosed CVD morbidity for each race and ethnicity.

**Results:**

We found excess CVD morbidity among non-Hispanic Black and Hispanic people experiencing poverty and diagnosed with hypertension compared with their non-Hispanic White counterparts. Multivariate analysis found a higher prevalence of CVD among participants of all races and ethnicities who were experiencing poverty and among non-Hispanic White people who had less than a college education. In addition, age, hypertension, poverty, smoking, and weight were significant predictors of the prevalence of CVD morbidity among all racial and ethnic groups.

**Conclusion:**

Changes to interventions, policies, and research may be needed to address the effect of key indicators of health disparities and specific SDOH, such as poverty level, that intersect with hypertension and contribute to excess CVD morbidity among people of some racial and ethnic groups, particularly non-Hispanic Black and Hispanic populations.

**Summary**

**What is already known on this topic?**

Some racial and ethnic minority groups are disproportionately affected by cardiovascular disease (CVD). Hypertension is a major risk factor for CVD. The social determinants of health, such as poverty, affect the development and course of CVD.

**What is added by this report?**

We found excess CVD morbidity among non-Hispanic Black and Hispanic people experiencing poverty and diagnosed with hypertension compared with their non-Hispanic White counterparts.

**What are the implications for public health practice?**

Comprehensive, culturally tailored, multilevel approaches, interventions, and policies that incorporate the intersectionality of common risk factors for CVD morbidity (ie, hypertension and age) with poverty are warranted in public health practice.

## Introduction

Death certificate data from 2020 show that 931,558 people died from cardiovascular disease (CVD) in the US, making it the leading cause of death (1). Hypertension is a major risk factor for CVD that affects approximately 1 in 2 adults (2). It is often undiagnosed and uncontrolled (3). Health disparities in CVD related to uncontrolled hypertension exist among non-Hispanic White, non-Hispanic Black, and Hispanic groups (4,5). For example, in a 2021 study that used National Health and Nutrition Examination Survey (NHANES) data, non-Hispanic Black and Hispanic participants had a significantly lower prevalence of blood pressure control (39% and 40%, respectively) than their non-Hispanic White counterparts (49%). Findings on the prevalence of awareness and treatment of hypertension among these groups were discordant with findings on the prevalence of hypertension. Although the prevalence of hypertension awareness and treatment was similar among non-Hispanic Black and non-Hispanic White participants, the prevalence of hypertension was significantly higher among non-Hispanic Black participants (45.5% vs 31.4%). Conversely, the prevalence of hypertension awareness and treatment was lower among Hispanic participants than non-Hispanic White participants, but the 2 groups had a similar prevalence of hypertension (6). The higher prevalence of hypertension observed among non-Hispanic Black participants may disproportionately increase their risk of CVD (2).

Some demographic characteristics, such as age and sex, are well known to increase the risk for CVD (4). Less well-described are the effects of social determinants of health (SDOH) (eg, income, educational attainment, employment status, psychosocial factors including racism, and environmental factors) — all of which are key indicators of health disparities (7). US studies have shown that people in higher income groups experience lower rates of CVD events than people in low-income groups (8). Educational attainment was found to have an inverse relationship with the prevalence of CVD morbidity (9). The American Heart Association updated its approach to defining and quantifying cardiovascular health risk factors in a new publication, *Life’s Essential 8,* which incorporates the foundational role of SDOH to achieving optimal and equitable cardiovascular health (10).

The intersection of SDOH and hypertension can create health disparities and affect the prevalence of CVD morbidity. Specific SDOH may interact with hypertension to have a combined effect that leads to an excess burden of CVD morbidity (6). Additionally, SDOH may also negatively affect specific populations and lead to hypertension. Intersectionality, defined by the National Collaborating Centre for Healthy Public Policy, is a useful concept to address these risk factors for CVD (11). “Intersectionality is an approach or lens that recognizes that health is shaped by multi-dimensional overlapping of factors, such as race and ethnicity, class, income, education, age, ability, sexual orientation, immigration status, and geography” (11). For example, some data illustrate that multidimensional SDOH are factors strongly associated with a higher prevalence of hypertension (12). These multidimensional SDOH affect access to health care, quality of health care, and access to safe physical activity outlets and healthy foods, and they are related to higher levels of psychosocial stress (12).

Furthermore, people experiencing poverty and people who are financially and medically underresourced may be more likely than their wealthier and better-resourced counterparts to be exposed to psychosocial and socioeconomic stressors, discrimination, and racism, which can exacerbate the lack of hypertension control and increase CVD disparities (7,13). We examined the association of a single SDOH (poverty level) with diagnosed CVD morbidity and the relative excess risk due to interaction (RERI) of poverty level and hypertension on CVD morbidity among non-Hispanic Black, non-Hispanic White, and Hispanic adults aged 30 years or older.

## Methods

We analyzed 19 years (1999–2018) of data from NHANES. Because NHANES uses a complex, multistage, probability sampling design, we aggregated these data and applied proper sampling weights (14,15). Overall, 101,316 people participated in NHANES during this period. For this analysis, we included only people who were aged 30 years or older and reported annual income (n = 37,881). We excluded participants with missing values for blood pressure or CVD (n = 3,631). In addition, we excluded participants who were not Hispanic, non-Hispanic Black, or non-Hispanic White because of small sample sizes (n = 2,760). The final sample included 31,490 people aged 30 years or older, including 8,386 (26.6%) Hispanic, 6,821 (21.7%) non-Hispanic Black, and 16,283 (51.7%) non-Hispanic White participants.

We used the 2019 American College of Cardiology/American Heart Association Guidelines on the Primary Prevention of Cardiovascular Disease to define diagnosed CVD morbidity as self-reported coronary heart disease, angina, myocardial infarction, or stroke (16). Participants were asked in the home by trained interviewers, “Has a doctor or other health professional ever told you that you had [coronary heart disease/angina/myocardial infarction/stroke]?” During the NHANES clinical examinations, a trained clinician used an NHANES protocol for measuring blood pressure, which is based on methods developed by the American Heart Association (17). NHANES defines hypertension as having an average of 4 systolic blood pressure readings ≥140 mm Hg, an average of 4 diastolic blood pressure readings ≥90 mm Hg, or currently taking antihypertensive medication.

NHANES also collected information on age, height and weight (used to calculate body mass index [BMI]), and sex, during the NHANES clinical examination, and smoking status, poverty level, and educational attainment during the household interview (17). We categorized educational attainment into 4 ordinal groups for the multivariate analysis: less than high school diploma, high school diploma, some college, and college graduate. We categorized age into 6 ordinal groups: 30 to 44 years, 45 to 54 years, 55 to 64 years, 65 to 74 years, 75 to 79 years, and 80 years or older. Similarly, we categorized BMI (calculated as weight in kg divided by height in m^2^) into 6 ordinal groups: underweight (BMI < 18.5), normal (18.5 ≤BMI <25.0), overweight (25.0 ≤BMI <30.0), obese class I (30.0 ≤BMI <35.0), obese class II (35.0 ≤BMI <40.0), and obese class III (BMI ≥40.0) (18). Smoking status was categorized into current (smoke every day or some days), former (have smoked at least 100 cigarettes in their lifetime), or never smokers. Poverty level was determined by the ratio of family income to poverty guidelines from the US Census Bureau’s current official poverty thresholds (17). If the ratio was less than 1, then the individual was assigned as being in poverty; if the ratio was 1 or more, then the individual was designated as not being in poverty.

Statistical analyses were treated as complete case analyses, where models excluded people with missing information. We chose specific race and ethnicity models because of evidence of inequities in the distribution of SDOH that may differentially affect CVD in certain race and ethnicity groups, especially the non-Hispanic Black population (19), and to address and control for any covariates that may share a relationship with race and ethnicity. We used a survey-based generalized linear model, which used a Poisson family and log link, to obtain relative risks (RRs) (20). The models calculating RRs for diagnosed CVD morbidity controlled for age, BMI, hypertension status, poverty level, educational attainment, sex, and smoking status. Importantly, we left age and BMI as continuous in these models.

We explored the interaction between poverty level with hypertension on diagnosed CVD morbidity on an additive scale for each race and ethnicity. Analyzing interactions on the additive scale is more applicable to public health than studying multiplicative interactions (21). This interaction was calculated by using a distinct measure known as the relative excess risk due to interaction (RERI) (21):

RERI = (RR_11_ − RR_10_ − RR_01_ + 1)

where RR_11_ is the relative risk of having CVD with the presence of SDOH and hypertension, RR_10_ is the relative risk of having CVD with the presence of only the SDOH, and RR_01_ is the relative risk of having CVD with the presence of only hypertension.

RERI is a measure that examines the difference in relative risk between the sum of the individual exposures and the joint exposure. RERI values greater than 0 signify a synergistic interaction, where the result is amplified by the interaction. Conversely, RERI values less than 0 signify an antagonistic interaction, where the result is diminished by the interaction. The models used to calculate RERI controlled for age, BMI, sex, and smoking status. Additionally, we used weighted frequencies from PROC SURVEYFREQ (SAS Institute Inc) to assess the CVD prevalence per 100,000 people among Hispanic, non-Hispanic Black, and non-Hispanic White populations. We stratified each prevalence of CVD by hypertension status and poverty level. We then calculated the expected prevalence of CVD among people with hypertension and poverty (Equation 1) and compared the expected prevalence with the observed prevalence among people with hypertension and poverty to assess the excess burden (22).

Equation (1) Expected Prev_H + P +_ = Observed Prev_H + P −_ + Observed Prev_H − P +_ − Observed Prev_H − P −_

Equation (2) Excess Burden = Observed Prev_H + P +_ − Expected Prev_H + P +_

Additionally, in our descriptive analysis of the characteristics of our study sample, we used PROC SURVEYFREQ and PROC SURVEYMEANS (SAS Institute Inc) to obtain frequencies and means. Statistical significance for all analyses was determined at *P* < .05. We performed statistical analyses in SAS software version 9.4 (SAS Institute Inc), *survey* package version 4.2 in R (23), and *interactionR* package in R (24). This study did not use data containing personally identifiable information; therefore, institutional review board assessment was not necessary per the policy of the Centers for Disease Control and Prevention.

## Results

Overall, Hispanic participants in our sample were younger than non-Hispanic Black participants and non-Hispanic White participants: 50.4% of Hispanic participants, 40.4% of non-Hispanic Black participants, and 31.9% of non-Hispanic White participants were aged 30 to 44 years (Table 1). A larger percentage of non-Hispanic Black participants were in obesity classes I, II, and III (46.8%) than were Hispanic (40.4%) and non-Hispanic White (35.9%) participants. Non-Hispanic Black participants had the highest prevalence of hypertension (48.8%) compared with non-Hispanic White (37.6%) and Hispanic (27.9%) participants. When comparing poverty level by race and ethnicity, Hispanic participants had the highest percentage of people living in poverty (26.1%) compared with non-Hispanic Black (21.4%) and non-Hispanic White (7.5%) participants. A higher percentage of Hispanic participants had less than a high school diploma (43.2%) compared with non-Hispanic Black (25.0%) and non-Hispanic White (11.7%) participants. Both non-Hispanic White and non-Hispanic Black participants had the same prevalence of diagnosed CVD (11.6%), which was higher than the prevalence among Hispanic participants (6.9%).

**Table 1:** Characteristics of National Health and Nutrition Examination Survey Population of Adults Aged ≥30 Years, 1999–2018

Characteristic	No. (weighted column %)^a^^,^ b		
	Hispanic	Non-Hispanic Black	Non-Hispanic White
**Age, mean (SE), y**	47.0 (0.3)	49.9 (0.2)	53.4 (0.2)
**Age group, y**			
30–44	2,973 (50.4)	2,179 (40.4)	4,516 (31.9)
45–54	1,727 (23.8)	1,437 (25.2)	3,037 (24.1)
55–64	1,768 (13.8)	1,512 (18.1)	2,731 (19.3)
65–74	1,346 (8.3)	1,082 (10.1)	2,743 (14.1)
75–79	299 (1.9)	326 (3.3)	1,139 (4.6)
≥80	273 (1.8)	285 (2.9)	2,117 (6.0)
**BMI, mean (SE)^c^**	29.8 (0.12)	30.8 (0.12)	28.9 (0.08)
**Weight status^d^**			
Underweight	149 (1.7)	223 (2.8)	533 (2.6)
Normal	1,506 (18.3)	1,399 (20.2)	4,485 (27.6)
Overweight	3,304 (39.5)	2,068 (30.1)	5,574 (33.9)
Obese class I	2,101 (24.4)	1,552 (22.6)	3,300 (20.8)
Obese class II	851 (10.2)	856 (13.0)	1,450 (9.1)
Obese class III	475 (5.8)	723 (11.2)	941 (6.0)
**Education^e^**			
Less than high school diploma	4,351 (43.2)	1,874 (25.0)	2,683 (11.7)
High school diploma	1,495 (20.8)	1,661 (24.7)	4,271 (25.0)
Some college	1,666 (23.1)	2,105 (31.7)	4,870 (30.8)
College graduate	863 (12.8)	1,168 (18.4)	4,447 (32.5)
**Cardiovascular disease^f^**			
Yes	832 (6.9)	946 (11.6)	2,645 (11.6)
No	7,554 (93.1)	5,875 (88.4)	13,638 (88.4)
**Hypertension**			
Yes	3,096 (27.9)	3,740 (48.8)	7,072 (37.6)
No	5,290 (72.1)	3,081 (51.2)	9,211 (62.4)
**Poverty^g^**			
Yes	2,276 (26.1)	1,422 (21.4)	1,871 (7.5)
No	5,763 (73.9)	5,162 (78.6.)	14,003 (92.5)
**Sex**			
Male	3,978 (49.8)	3,290 (44.1)	8,173 (48.3)
Female	4,408 (50.2)	3,531 (55.9)	8,110 (51.7)
**Smoking^h^**			
Current	1,330 (17.7)	1,730 (25.9)	3,394 (19.9)
Former	2,161 (22.9)	1,485 (18.2)	5,395 (30.6)
Never	4,890 (59.4)	3,600 (55.9)	7,486 (49.5)

Abbreviation: BMI, body mass index.

a Values are number (weighted column %) unless otherwise indicated.

b Differences between 3 racial and ethnic groups were determined by χ^2^ tests and analysis of variance; *P* < .001 for all.

c Calculated as weight in kilograms divided by height in meters squared.

d Categories established by the Centers for Disease Control and Prevention (18). BMI <18.5, underweight; BMI 18.5 to <25.0, normal; BMI 25.0 to <30.0, overweight; BMI ≥30.0, obesity. Obese class I, 30.0 ≤ BMI <35.0; obese class II, 35.0 ≤ BMI <40.0; obese class III, BMI ≥40.0.

e 36 participants were missing information on educational attainment.

f Participants were asked in the home by trained NHANES interviewers, “Has a doctor or other health professional ever told you that you had [coronary heart disease/angina/myocardial infarction/stroke]?”

g 993 participants were missing income information; poverty level could not be determined.

h 19 participants were missing information on smoking.

The prevalence of diagnosed CVD morbidity increased significantly by age among all race and ethnicity groups. Rates of high BMI, hypertension, poverty, and smoking were also significantly associated with diagnosed CVD morbidity (Table 2). Sex and educational attainment were significantly associated with diagnosed CVD morbidity among non-Hispanic White participants but not among non-Hispanic Black and Hispanic participants (Table 2).

**Table 2:** Adjusted Relative Risks^a^ of Ever Having Diagnosed Cardiovascular Disease Among the National Health and Nutrition Examination Survey Population of Adults Aged ≥30 Years, 1999–2018

Characteristic	Relative risk (95% CI)^a^		
	Hispanic	Non-Hispanic Black	Non-Hispanic White
**Age**	1.05 (1.04–1.06)	1.04 (1.03–1.04)	1.06 (1.05–1.06)
**Body mass index**	1.02 (1.01–1.04)	1.02 (1.02–1.03)	1.03 (1.02–1.04)
**Hypertension**	1.86 (1.55–2.23)	2.20 (1.80–2.69)	1.50 (1.34–1.67)
**Poverty**	1.44 (1.19–1.73)	1.51 (1.32–1.72)	1.53 (1.33–1.75)
**Male vs female**	1.05 (0.87–1.28)	1.05 (0.90–1.24)	1.41 (1.27–1.57)
**Education**			
Less than high school diploma vs high school diploma	1.11 (0.84–1.46)	1.05 (0.88–1.25)	1.14 (1.02–1.28)
Some college vs high school diploma	1.24 (0.90–1.71)	1.09 (0.90–1.32)	0.86 (0.77–0.95)
College graduate vs high school diploma	0.77 (0.50–1.20)	0.93 (0.72–1.22)	0.70 (0.61–0.80)
**Smoking**			
Current vs never	1.70 (1.29–2.24)	1.74 (1.48–2.04)	1.93 (1.70–2.20)
Former vs never	1.30 (1.04–1.64)	1.48 (1.26–1.74)	1.34 (1.22–1.48)

a All relative risks were adjusted for all indicators in each race- and ethnicity-specific column.

The association between hypertension and diagnosed CVD morbidity was significantly modified by poverty level among Hispanic participants, after controlling for age, BMI, sex, and smoking status (Table 3). The RERI was 0.53 (−0.08 to 1.15) among Hispanic participants, 0.38 (−0.30 to 1.07) among non-Hispanic Black participants, and −0.02 (−0.58 to 0.55) among non-Hispanic White participants.

**Table 3:** Additive Interaction as Measured by RERI of Poverty With and Without Hypertension on Prevalence of Diagnosed Cardiovascular Disease Among the NHANES Population of Adults Aged ≥30 Years, 1999–2018

Category	Hispanic^a^	Non-Hispanic Black^a^	Non-Hispanic White^a^
Relative risk (95% CI) for hypertension only	1.82 (1.47 to 2.27)	2.29 (1.84 to 2.85)	1.56 (1.40 to 1.74)
Relative risk (95% CI) for poverty only	1.41 (1.07 to 1.84)	1.72 (1.24 to 2.38)	1.90 (1.51 to 2.39)
Relative risk (95% CI) for both poverty and hypertension	2.76 (2.14 to 3.57)	3.39 (2.66 to 4.32)	2.44 (2.02 to 2.96)
RERI (95% CI)^b^	0.53 (−0.08 to 1.15)	0.38 (−0.30 to 1.07)	−0.02 (−0.58 to 0.55)

Abbreviations: NHANES, National Health and Nutrition Examination Survey; RERI, relative excess risk due to interaction.

a Controlling for age, body mass index, sex, and smoking status.

b RERI values greater than 0 signify a synergistic interaction, where the result is amplified by the interaction. Conversely, RERI values less than 0 signify an antagonistic interaction, where the result is diminished by the interaction.

The excess burden for diagnosed CVD per 100,000 individuals from the interaction between hypertension and poverty was calculated as 4,668 cases among non-Hispanic White, 5,922 among non-Hispanic Black, and 7,952 among Hispanic people (Table 4). The additive interaction for non-Hispanic White people was approximately 0 (Table 3), which may indicate that the excess burden of 4,668 per 100,000 individuals may be due to external sources. However, the excess burden, due to an additive interaction among non-Hispanic Black and Hispanic groups, was clinically relevant. This excess burden of CVD indicates that approximately 6,000 and 8,000 cases per 100,000 individuals among non-Hispanic Black individuals and Hispanic individuals may be developing CVD in excess because of the additive interaction’s presence outside the individual exposure that hypertension and poverty have on the development of CVD.

**Table 4:** Excess Burden of Poverty and Hypertension on Prevalence of Diagnosed Cardiovascular Disease per 100,000 People Among the NHANES Population of Adults Aged ≥30 Years, 1999–2018

Poverty level^a^	No. of cases					
	Hispanic		Non-Hispanic Black		Non-Hispanic White	
	No hypertension	Hypertension	No hypertension	Hypertension	No hypertension	Hypertension
Not in poverty	3,472	12,112	4,124	16,668	6,027	19,390
In poverty	4,980	21,572	7,610	26,076	11,538	29,569
Excess burden	—	7,952	—	5,922	—	4,668

Abbreviation: — does not apply.

a Poverty level was determined by the ratio of family income to poverty guidelines from the US Census Bureau’s current official poverty thresholds (17). If the ratio was less than 1, then the individual was assigned as being in poverty; if the ratio was 1 or more, then the individual was designated as not being in poverty.

## Discussion

This study compiled 19 years of NHANES data and analyzed the combined exposure of poverty and hypertension on the prevalence of CVD that exceeded the expected prevalence of CVD among Hispanic, non-Hispanic Black, and non-Hispanic White participants. One key finding is that poverty level was independently associated with increased risk for diagnosed CVD morbidity among all 3 racial and ethnic groups studied. This finding confirms the findings by Abdalla and colleagues, who used NHANES data from 1999–2016 and found a greater prevalence of diagnosed CVD morbidity among lower-income groups than higher-income groups (25). In addition, low educational attainment (ie, less than college education) was found to be a strong predictor of CVD among non-Hispanic White participants but not among non-Hispanic Black and Hispanic participants. Previous studies support that low educational attainment may be associated with CVD because of behavioral characteristics, such as smoking, lack of physical activity, and obesity, in addition to other SDOH (26).

It is well established that certain populations experience health disparities and are disproportionately affected by CVD and hypertension (2,3,5,27). Additionally, a working poor segment of the US population meets the poverty–income ratio or poverty threshold; they often do not have health insurance and are not eligible for federal assistance programs such as the Supplemental Nutrition Assistance Program (28). Our results show that one key SDOH, poverty, may interact with hypertension to influence diagnosed CVD morbidity disproportionately among certain groups. This result is exemplified by the larger additive interaction of poverty and hypertension among non-Hispanic Black and Hispanic people, compared with non-Hispanic White people. Although not statistically significant, the larger additive interaction among non-Hispanic Black and Hispanic people is noteworthy and clinically relevant. This excess burden of diagnosed CVD morbidity among non-Hispanic Black and Hispanic people, resulting from the intersection of poverty, race and ethnicity, and hypertension may lead to health disparities. Similar results were found by a study that analyzed the risk of hypertension by income, racial and ethnic composition, and geographic location (29). The study showed that non-Hispanic White participants with higher incomes had significantly lower odds of hypertension than non-Hispanic Black participants with low incomes (24% vs 52%) (29).

Observable SDOH, such as poverty, are influenced by numerous factors at multiple levels (10,30). SDOH include all aspects of life, such as the places where people live, work, and play (31,32). If the public health and medical communities are to reduce the equity gap in CVD among certain populations (ie, race and ethnicity or low income), then the convergence of upstream psychosocial and socioeconomic stressors, such as poverty, depression, job stress, financial stress, segregated neighborhoods, and neighborhood poverty level, should be considered (31–33). Furthermore, efforts designed to reduce disparities in the prevalence of diagnosed CVD morbidity may need to address the convergence of these multilevel risk factors. These SDOH are modifiable. In a scenario where the negative effects of an SDOH can be alleviated, the prevalence would decrease not only by the SDOH’s independent effect but also by its excess morbidity with hypertension. By analyzing the additive interaction and assessing the excess morbidity, public health and policy officials can intuitively see the ramifications of any potential intervention that disrupts the interaction. These ramifications can be seen intuitively because the assessment of an additive interaction is performed in an absolute manner rather than by interpreting the results of multiplicative interactions, which are relative to one another.

Other factors and stressors — such as discrimination and racism — may be considered. A study that examined the relationship between blood pressure and self-reported racial discrimination and responses to unfair treatment found a significant association between hypertension and racial discrimination (33). Characteristics historically linked to racism, discrimination, and exclusion are factors that may be incorporated into interventions to address inequities in the prevalence of CVD morbidity. Public health interventions that also address racism may need to be considered to meet the diverse needs of populations who are affected by racism and are medically underserved. The impact of centuries of racism is pervasive, deeply embedded in our society, and has a negative effect on racial and ethnic minority populations (32,33). The potentially strong repercussions of discrimination, racism, and health care disparities resulting from treatment or implicit bias (ie, people receiving inadequate treatment of hypertension because of language barriers or their race or ethnicity) are important factors that could be considered. Racism and discrimination may play a role as a stressor and determinant of health that detrimentally affects blood pressure and heart health for non-Hispanic Black people in the US.

*The Surgeon General’s Call to Action on Hypertension* describes the historical impact of racial discrimination, identifies SDOH as the “third arm of risk,” and expresses the need to equitably distribute human, technical, and financial resources to address these factors (29). Furthermore, in its 2010 report, *A Population-based Policy and Systems Change Approach to Prevent and Control Hypertension*, the Institute of Medicine suggests integrating hypertension prevention and control interventions into the policies and programs of public health practices in ways that support healthy eating, active living, and obesity, as well as focus on populations that are most likely to be affected by hypertension (32). In addition, population-based policy interventions focusing on systems-level improvements in SDOH, such as poverty, food insecurity, low educational attainment, and language barriers, may be considered when seeking ways to address disparities in the prevalence of CVD morbidity.

### Limitations

Our study has potential limitations. Selection bias and the selected SDOH affected the study population. For example, some racial and ethnic minority groups (eg, American Indian, Alaska Native, Asian) were excluded from our analysis because of small sample sizes. Additionally, self-reported survey data, residual confounding in the analysis, and survey response rates may have affected study outcomes. The measure for diagnosed CVD morbidity in our study was affected by survivor bias: people who did not survive a myocardial infarction or stroke would not have participated in NHANES. Furthermore, because the study was cross-sectional, causation cannot be determined, which limits possible interpretations. Observational studies that use effective methods for examining causation are required to establish a hypothesis suggesting causal inference.

### Conclusion

Our findings reinforce the effect that SDOH, such as poverty, have on the prevalence of CVD among Hispanic, non-Hispanic Black, and non-Hispanic White people with hypertension. Our results suggest that multilevel and multifaceted approaches could play a role in addressing hypertension and CVD prevention among some racial and ethnic groups. These approaches may include interventions, policies, and research to address the factors that contribute to inequities in CVD morbidity. Our sensitivity analyses showed that the addition of age as a covariate shifted the 95% CIs of RERIs toward the null. This interplay of poverty, hypertension, and age on CVD morbidity should be further investigated as factors that may exacerbate the disparities in CVD morbidity, especially within a temporal context. Intersectionality can be used as a framework to guide future studies and interventions. This study, which included 19 years of NHANES data, may help to advance public health practice, generate new research ideas, and influence clinical practice by contributing to the knowledge base in the areas of CVD morbidity, hypertension, SDOH, health inequities, and health disparities.
